# Serologic Evidence of Fruit Bat Exposure to Filoviruses, Singapore, 2011–2016

**DOI:** 10.3201/eid2401.170401

**Published:** 2018-01

**Authors:** Eric D. Laing, Ian H. Mendenhall, Martin Linster, Dolyce H. W. Low, Yihui Chen, Lianying Yan, Spencer L. Sterling, Sophie Borthwick, Erica Sena Neves, Julia S. L. Lim, Maggie Skiles, Benjamin P. Y.-H. Lee, Lin-Fa Wang, Christopher C. Broder, Gavin J. D. Smith

**Affiliations:** Uniformed Services University, Bethesda, Maryland, USA (E.D. Laing, L. Yan, S.L. Sterling, C.C. Broder);; Duke-National University of Singapore Medical School, Singapore, Singapore (I.H. Mendenhall, M. Linster, D.H.W. Low, Y. Chen, S. Borthwick, E.S. Neves, J.S.L. Lim, L.-F. Wang, G.J.D. Smith);; North Carolina State University, Raleigh, North Carolina, USA (M. Skiles);; National Parks Board, Singapore (B.P.Y.-H. Lee);; Duke University, Durham, North Carolina, USA (L.-F. Wang, G.J.D. Smith)

**Keywords:** virus surveillance, serology, Southeast Asia, Ebola virus, Bundibugyo virus, Sudan virus, virus envelope glycoprotein, viruses, filoviruses, fruit bats, Singapore

## Abstract

To determine whether fruit bats in Singapore have been exposed to filoviruses, we screened 409 serum samples from bats of 3 species by using a multiplex assay that detects antibodies against filoviruses. Positive samples reacted with glycoproteins from Bundibugyo, Ebola, and Sudan viruses, indicating filovirus circulation among bats in Southeast Asia.

The genus *Ebolavirus* comprises 5 virus species: *Zaire ebolavirus* (EBOV), *Sudan ebolavirus* (SUDV), *Bundibugyo* *ebolavirus* (BDBV), *Taï Forest* *ebolavirus* (TAFV), and *Reston ebolavirus* (RESTV). The genus *Marburgvirus* comprises 1 species, *Marburg marburgvirus*, which includes 2 closely related virus strains: Marburg virus (MARV) and Ravn virus (RAVV). Viruses within the *Ebolavirus* and *Marburgvirus* genera are zoonotic; EBOV was the causative agent of the 2014–2016 Ebola virus disease epidemic in West Africa (*1*). *Rousettus* bats in Africa have been identified as *Marburgvirus* hosts (*2*), and viral nucleic acid and serologic evidence suggests that bats are also natural hosts of* Ebolavirus* spp. (*3*). Yet it remains unclear which species are the definitive reservoirs of filoviruses.

Ecologic models of *Ebolavirus* and *Marburgvirus* geographic distribution and habitat ranges of potential reservoir bat species suggest that both groups are distributed throughout Asia (*3*,*4*). Serologic evidence of filoviruses in frugivorous bats in Bangladesh, China, and the Philippines has been reported (*5*–*7*), and RESTV nucleic acid was detected in an insectivorous bat in the Philippines, where RESTV is considered endemic (*8*). We examined pteropodid bats of 3 species: *Cynopterus brachyotis*, *Eonycteris spelaea*, and *Penthetor lucasi*, which are widely distributed across Southeast Asia and share ecologic niches (*9*).

## The Study

During 2011–2016, we collected serum from bats of the 3 aforementioned species in Singapore and screened samples for evidence of exposure to filoviruses. Samples were collected with permission from the National University of Singapore Institutional Animal Care and Use Committee (B01/12) and the National Parks Board (NP/RP11–011–3a). We diluted venous blood 1:10 in phosphate-buffered saline and then centrifuged, recovered, and heat-inactivated the serum at 56°C for 30 minutes and stored it at −80°C.

We developed a Bio-Plex (Bio-Rad, Hercules, CA, USA) bead-based multiplex assay that simultaneously probes serum for immunoglobulins specific to the viral envelope glycoproteins (GPs) from representative strains of all described *Ebolavirus* and *Marburgvirus* species (Table 1). A human FreeStyle 293-F stable cell-line expression system was used to produce the *Ebolavirus* and *Marburgvirus* spp. GPs as a soluble GP consisting of the entire ectodomain, sGP_(1,2)_, which retains a native-like oligomeric conformation, as described previously with modifications (*10*). In brief, each GP_(1,2)_ coding sequence was truncated at the C-terminus to remove the predicted transmembrane domain and cytoplasmic tail, then appended with the GCN trimerization peptide sequence (*10*) together with a factor Xa protease cleave site and a Twin-Strep-tag sequence (IBA Lifesciences, Göttingen, Germany). The sGP_(1,2)_ proteins were produced in serum-free conditions and purified by Strep-Tactin XT technology (IBA Lifesciences). The Twin-Strep-tag was removed by factor Xa enzymatic cleavage; factor Xa was removed by Xarrest Agarose (Merck Millipore, Billerica, MA, USA); sGP_(1,2)_ was purified further by S-200 size exclusion chromatography, concentrated, and stored frozen. These sGP_(1,2)_s were coupled to carboxylated beads (Bio-Rad). Screening was performed on a Bio-Rad Bio-Plex 200.

**Table 1:** *Ebolavirus* and *Marburgvirus* species soluble envelope glycoproteins conjugated Bio-Plex beads used in multiplex assay to detect antibodies against filoviruses*

Virus	Isolation host/location	Bio-Plex bead no.	NCBI accession no.
Ebola virus/H.sapiens/COD/1976/Yambuku-Mayinga	Human/DRC	33	NC_002549.1
Bundibugyo virus/H. sapiens/UGA/2007	Human/Uganda	64	FJ217161.1
Taï Forest virus/H. sapiens/COV/1994/Pauleoula-CI	Human/Côte d'Ivoire	57	NC_014372
Sudan virus/H. sapiens/UGA/2000/Gulu-808892	Human/Uganda	77	NC_006432.1
Reston virus/M. fascicularis/USA/1989/Pennsylvania	Macaque/USA	85	AF522874.1
Reston virus/S. domesticus/PHL/2008/Reston08-A	Swine/Philippines	72	FJ621583.1
Marburg virus/H. sapiens/KEN/1980/Musoke	Human/Kenya	37	Z12132 S55429
Marburg virus/H. sapiens/AGO/2005/Ang0126	Human/Angola	28	DQ447656.1
Ravn virus/H. sapiens/KEN/1987/Kitum cave-810040	Human/Kenya	49	NC_024781.1

*Bio-Plex manufactured by Bio-Rad (Hercules, CA, USA). DRC, Democratic Republic of the Congo; NCBI, National Center for Biotechnology Information.

In the absence of confirmed filovirus-negative bat serum, we used methods developed by Peel et al. to establish a median fluorescence intensity (MFI) cutoff value (*11*). We confirmed a cutoff value of 200 MFI (Technical Appendix), as was previously used for *Eidolon helvum* bat serum in a Bio-Plex serologic assay (*12*). We screened 409 samples with our *Ebolavirus* and *Marburgvirus* spp. sGP_(1,2)_ Bio-Plex assay modified from that described by Bossart et al. (*13*). Samples were diluted 1:100 and tested in duplicate; the sGP_(1,2)_-coupled beads were mixed with individual samples; and a 1:1 combination of recombinant biotinylated-protein A/protein G (1:500) (Pierce, Rockford, IL, USA) was added to the wells, followed by addition of streptavidin-phycoerythrin (1:1,000) (Bio-Rad) and determination of MFI.

**Figure 1:**
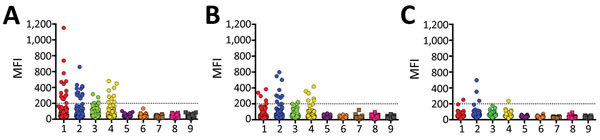
Mean fluorescence intensity (MFI) values obtained from Bio-Plex assay (Bio-Rad, Hercules, CA, USA) screening of individual serum samples from bats of 3 species with soluble filovirus glycoproteins. Dashed line indicates the cutoff value at 200 MFI. 1, *Zaire ebolavirus*; 2, *Bundibugyo ebolavirus*; 3, *Taï Forest ebolavirus*; 4, *Sudan ebolavirus*; 5, *Reston ebolavirus*–monkey; 6, *Reston ebolavirus*–pig; 7, Marburg virus–Musoke; 8, Marburg virus–Angola; 9, Ravn virus.

Samples were positive for 17 (9.1%) of 186 *E. spelaea*, 13 (8.5%) of 153 *C. brachyotis*, and 3 (4.3%) of 70 *P. lucasi* bats (Figure 1). Positive samples reacted with EBOV, BDBV, SUDV, or TAFV sGP_(1,2)_. However, no samples were positive for RESTV, MARV, or RAVV sGP_(1,2)_. We further examined positive samples to determine cross-reactivity between the *Ebolavirus* spp. sGP_(1,2)_ (Table 2). Twelve (71%) samples from *E. spelaea* bats cross-reacted with >2 *Ebolavirus* spp. sGP_(1,2)_ (BDBV, EBOV, SUDV, or TAFV). In contrast, 8 (62%) *C. brachyotis* and 2 (66%) *P. lucasi* samples were positive for only 1 sGP_(1,2)_ (BDBV or SUDV).

**Table 2:** Bio-Plex median fluorescence intensity values for bat serum positive for >1 filovirus antigen*

Bat species, ID	EBOV	BDBV	TAFV	SUDV	RESTVm	RESTVp	MARV(Mus)	MARV(Ang)	RAVV
*Eonycteris spelaea*, n = 186									
0805149†	**738**	124	68	40	44	22	23	21	24
080814	86	**318**	105	**258**	26	12	17	16	20
082154	143	161	113	**214**	35	41	21	31	39
052313	**284**	**408**	177	**285**	89	72	29	23	30
052335	**203**	191	124	**219**	42	21	38	38	24
052339	**357**	**306**	141	**293**	54	31	26	26	42
071839	**330**	**299**	164	**480**	65	44	28	33	45
071842	**446**	**327**	**202**	**362**	65	49	42	38	57
110733	126	**416**	166	95	58	42	34	42	58
011603†	**1151**	130	91	69	36	32	51	35	39
011616	**252**	294	168	175	32	49	47	29	50
011656	**306**	**386**	**204**	**394**	89	73	18	39	37
012309†	**579**	**659**	**315**	69	35	31	27	33	35
021303	**478**	**431**	188	**450**	52	37	24	30	47
111903	**469**	**384**	**276**	113	52	57	37	69	54
111907	**285**	**336**	**213**	158	39	36	29	50	30
042722	**260**	**262**	174	**1**67	75	31	54	24	42
*Cynopterus brachyotis*, n = 153									
051253	121	133	59	**242**	40	41	19	25	68
0516613	146	**293**	127	73	47	36	25	29	22
0516632	138	139	86	**356**	35	25	28	34	34
0726122†	119	**501**	100	60	40	46	25	19	29
1103241	84	141	128	**241**	50	47	66	38	34
100903	148	**201**	71	108	42	33	18	16	36
100914	74	**228**	70	55	39	38	30	27	26
100925	166	**304**	109	116	43	18	33	30	28
021357	**201**	**299**	179	**264**	65	44	25	55	47
050804	**242**	**276**	140	124	41	30	34	33	44
050818	**383**	**374**	198	**332**	60	55	29	26	68
040807†	**297**	**597**	194	192	40	38	122	95	32
042701†	**339**	**547**	**222**	**417**	60	78	54	25	62
*Penthetor lucasi*, n = 70									
062590†	34	**496**	93	39	36	18	23	17	23
070409†	95	**238**	129	89	62	27	34	36	37
112112†	**251**	**352**	148	**235**	51	29	23	23	29

**Figure 2:**
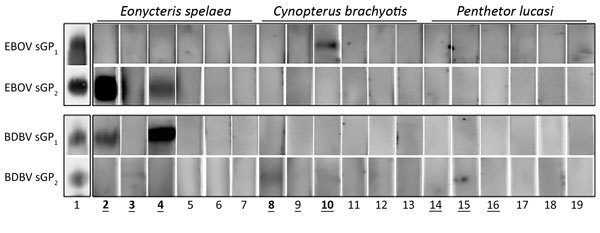
Western blot results of individual bat serum samples probed against *Zaire ebolavirus* and *Bundibugyo ebolavirus* glycoproteins 1 and 2 (GP_1_, GP_2_). Boldface indicates positivity by Western blot and underlining indicates positivity by Bio-Plex (Bio-Rad, Hercules, CA, USA). 1, soluble GP_1_ and GP_2_ blotted with control anti–Ebola virus nonhuman primate polyclonal serum that demonstrates cross-reactivity with *Bundibugyo ebolavirus* soluble GP. Other numbers along baseline correspond to the following sample identifiers, also used in Table 2: 2, 0805149; 3, 012309; 4, 011603; 5, 0116048; 6, 0719036; 7, 1128015; 8, 0726122; 9, 042701; 10, 040807; 11, 0512540; 12, 1009010; 13, 0408029; 14, 070409; 15, 112112; 16, 062590; 17, 0228004; 18, 0919025; 19, 0625095. BDBV, Bundibugyo virus; EBOV, Ebola virus.

*Bio-Plex manufactured by Bio-Rad (Hercules, CA, USA). Boldface indicates positive results. BDBV, Bundibugyo virus; EBOV, Ebola virus; ID, specimen identification number; MARV(Mus), Marburg virus–Musoke; MARV(Ang), Marburg virus–Angola; RESTVm, Reston virus–monkey; RESTVp, Reston virus–pig; SUDV, Sudan virus; RAVV, Ravn virus; TAFV, Taï Forest virus.  †Sample screened by Western blot and shown in Figure 2.

To further determine the cross-reactivity of positive samples and to corroborate Bio-Plex assay results for a selected number of samples, we performed Western blot (WB) assays (Figure 2). The filovirus GP_(1,2)_ is a trimer of heterodimeric GP_1_ and GP_2_ subunits. The trimeric-like sGP_(1,2)_ is the antigen in the multiplex Bio-Plex assay, whereas linearized monomeric sGP_1_ and sGP_2_ subunits are the antigens in WBs. Reduced and denatured EBOV or BDBV unconjugated sGP_(1,2)_ was loaded on 8% sodium dodecyl sulfate–polyacrylamide electrophoresis gels, transferred to a polyvinylidene difluoride membrane, and probed with 1:100 dilutions of positive and negative bat serum, as previously determined by the Bio-Plex assay. All 3 *E. spelaea* bat samples and 2 of 3 *C. brachyotis* bat samples that were Bio-Plex positive were also positive by WB and displayed reactivity with EBOV and BDBV GP_1_ and GP_2_ antigens; no *P. lucasi* bat samples positive by Bio-Plex were positive by WB.

## Conclusions

We present evidence of antibodies specific to filoviruses antigenically related to *Ebolavirus* spp. in 3 species of fruit bats widely distributed throughout Southeast Asia. We detected seroreactivity with *Ebolavirus* spp. but not *Marburgvirus* spp. GP. Despite the close relatedness of the viruses, we detected samples reacting with only SUDV, not RESTV, GP. This finding contrasts with previous reports of bat serum cross-reactivity with RESTV nucleoprotein (*5*,*7*,*14*). Possible explanations include 1) the fact that our customized Bio-Plex assay is based on conformational sGP_(1,2)_, which can differentiate antibody specificity better than the more sequence conserved nucleoprotein, and 2) the lack of evidence of RESTV GP positivity with *Cynopterus* and *Eonycteris* bat serum samples, which is in line with previous findings (both species were negative while only *Rousettus amplexicaudatus* bats were positive) (*7*). *E. spelaea* bats were previously predicted to be filovirus hosts (*15*), and sequences of novel filoviruses have been discovered in *E. spelaea* bat populations in Yunnan, China (*14*). Our data provide additional empirical evidence that populations of *C. brachyotis, E. spelaea,* and *P. lucasi* bats in Southeast Asia are hosts of filoviruses, which seem antigenically more closely related to EBOV, BDBV, and SUDV than to RESTV.

Examination of cross-reactivity of positive samples from *E. spelaea, C. brachyotis,* and *P. lucasi* bats revealed no clear patterns of preferential reactivity with EBOV, BDBV, or SUDV GP. Factors that might contribute to the lack of *P. lucasi* positivity by WB include sensitivity differences between Bio-Plex and WB assays paired with the change in sGP_(1,2)_ conformation. Two Bio-Plex EBOV-positive samples (*E. spelaea* samples 0805149 and 011603) reacted with EBOV sGP_2_ and BDBV sGP_1_ in the WB. Bio-Plex and WB data strongly suggest the presence of yet-undetected batborne filoviruses, which are antigenically related to but distinct from BDBV, EBOV, and SUDV circulating in local bat populations. Reasons why these filoviruses have remained undetected include their inability to cross the species barrier, the rarity of spillovers into humans or domestic animals, or the fact that spillover events cause mild or no disease. We suggest that a yet-undescribed diversity of filoviruses exists in Southeast Asia bat populations, a hypothesis supported by the recent identification of filovirus sequences in *E. spelaea* and *R. leschenaulti* bats in China (*14*,*16*). Comprehensive surveillance including serology and detection of viral nucleic acid, along with virus isolation, will help elucidate the characteristics of filoviruses endemic to Asia and identify bat species that function as maintenance populations and reservoirs.

Technical Appendix Median fluorescence intensity cutoff value determination and results for filoviruses in serum from fruit bats, Singapore, 2011–2016.
