# Supplementary material for: Serologic Evidence of Fruit Bat Exposure to Filoviruses, Singapore, 2011–2016
Source: Emerg Infect Dis. 2018 Jan;24(1):122–6. doi: 10.3201/eid2401.170401 (PMC5749470; doi:10.3201/eid2401.170401)
Supplement: Technical Appendix — Median fluorescence intensity cutoff value determination and results for filoviruses in serum from fruit bats, Singapore, 2011–2016. [file 17-0401-Techapp-s1.pdf]

Article DOI: https://doi.org/10.3201/eid2401.170401

Serologic Evidence of Fruit Bat Exposure to Filoviruses, Singapore, 2011–2016

Technical Appendix

Determination of Median Fluorescence Intensity (MFI) Cutoff Value

A Bayesian mixture model was first fitted to the data for individual glycoproteins within each bat species using Markov chain Monte Carlo. The model was implemented in R (*1*) with code provided by Alison Peel (*2*) that was adapted for use with the software packages JAGS (*3*) and rjags (*4*). The parameters of the fit were then used to calculate the MFI at which the p value of being seronegative was 0.001, as well as the p value at an MFI of 200 (Figure).

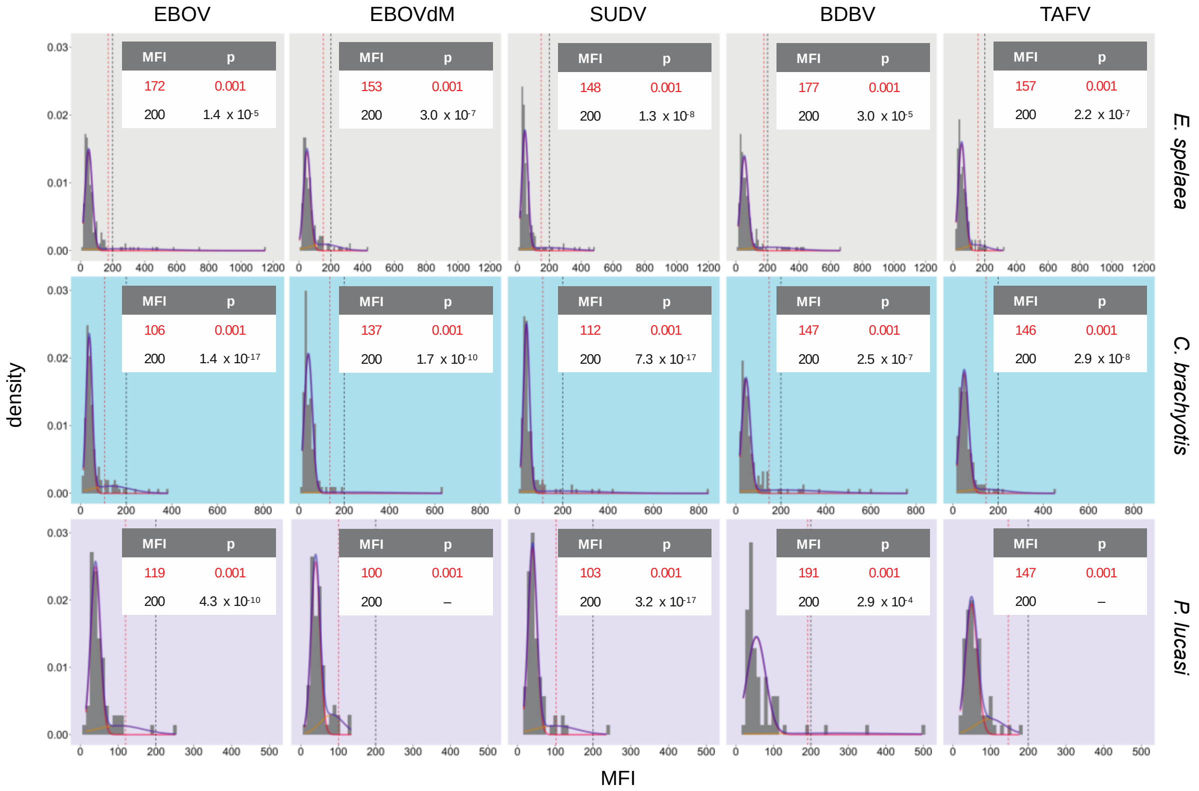

Figure. Median fluorescence intensity (MFI).

References

1. R Core Team,2016. R: A language and environment for statistical computing. R Foundation for Statistical Computing, Vienna. https://www.R-project.org/

2. Peel AJ, McKinley TJ, Baker KS, Barr JA, Crameri G, Hayman DTS, et al. Use of cross-reactive serological assays for detecting novel pathogens in wildlife: assessing an appropriate cutoff for henipavirus assays in African bats. J Virol Methods. 2013;193:295–303. PubMed http://dx.doi.org/10.1016/j.jviromet.2013.06.030

3. Plummer M, 2003. JAGS: A program for analysis of Bayesian graphical models using Gibbs sampling. http://citeseer.ist.psu.edu/plummer03jags.html

4.Plummer M, 2016. rjags: Bayesian Graphical Models using MCMC. R package version 4–6. https://CRAN.R-project.org/package=rjags
